# Preparing for human papillomavirus vaccine introduction in Kenya: implications from focus-group and interview discussions with caregivers and opinion leaders in Western Kenya

**DOI:** 10.1186/1471-2458-14-855

**Published:** 2014-08-16

**Authors:** Allison L Friedman, Kelvin O Oruko, Melissa A Habel, Jessie Ford, Jennine Kinsey, Frank Odhiambo, Penelope A Phillips-Howard, Susan A Wang, Tabu Collins, Kayla F Laserson, Eileen F Dunne

**Affiliations:** U.S. Centers for Disease Control & Prevention (CDC), 1600 Clifton Road, NE, 30333 Atlanta, GA USA; Department of Sociology, New York University, 295 Lafayette Street, 4th Floor, 10012-9605 New York, NY USA; Kenya Medical Research Institute (KEMRI)/CDC Research and Public Health Collaboration and CDC Center for Global Health, KEMRI Kisian Campus, PO Box 1578–40100, Kisumu, Kenya; Liverpool School of Tropical Medicine, Pembroke Place, L3 5QA Liverpool, UK; Department of Immunization, Vaccines and Biologicals, World Health Organization, Avenue Appia 20, 1211 27 Geneva, Switzerland; Kenya Ministry of Health, P.O. Box 30016–00100, Cathedral Rd, Nairobi, Kenya

**Keywords:** HPV vaccine acceptability, Qualitative research, Communication, Mobilization, Low-or-middle income countries, Kenya

## Abstract

**Background:**

Cervical cancer claims the lives of 275,000 women each year; most of these deaths occur in low-or middle-income countries. In Kenya, cervical cancer is the leading cause of cancer-related mortality among women of reproductive age. Kenya’s Ministry of Public Health and Sanitation has developed a comprehensive strategy to prevent cervical cancer, which includes plans for vaccinating preteen girls against human papillomavirus (HPV) by 2015. To identify HPV vaccine communication and mobilization needs, this research sought to understand HPV vaccine-related perceptions and concerns of male and female caregivers and community leaders in four rural communities of western Kenya.

**Methods:**

We conducted five focus groups with caregivers (n = 56) and 12 key-informant interviews with opinion leaders to explore cervical cancer-related knowledge, attitudes and beliefs, as well as acceptability of HPV vaccination for 9–12 year-old girls. Four researchers independently reviewed the data and developed codes based on questions in interview guides and topics that emerged organically, before comparing and reconciling results through a group consensus process.

**Results:**

Cervical cancer was not commonly recognized, though it was understood generally in terms of its symptoms. By association with cancer and genital/reproductive organs, cervical cancer was feared and stigmatized. Overall acceptability of a vaccine that prevents cervical cancer was high, so long as it was endorsed by trusted agencies and communities were sensitized first. Some concerns emerged related to vaccine safety (e.g., impact on fertility), program intent, and health equity.

**Conclusion:**

For successful vaccine introduction in Kenya, there is a need for communication and mobilization efforts to raise cervical cancer awareness; prompt demand for vaccination; address health equity concerns and stigma; and minimize potential resistance. Visible endorsement by government leaders and community influencers can provide reassurance of the vaccine’s safety, efficacy and benefits for girls and communities. Involvement of community leadership, parents and champions may also be critical for combatting stigma and making cervical cancer relevant to Kenyan communities. These findings underscore the need for adequate planning and resources for information, education and communication prior to vaccine introduction. Specific recommendations for communication and social-marketing strategies are made.

## Background

Each year, nearly 530,000 women develop cervical cancer and almost 270,000 die from it worldwide [1]. Most of these deaths occur in low- or middle-income countries (LMIC) with limited or no cervical-cancer screening programs. Kenya has one of the highest rates of cervical cancer in the world [2]. Among Kenyan women of reproductive age, cervical cancer is the most common cancer diagnosed and the leading cause of cancer-related mortality [3]. Kenya has developed a comprehensive strategy to address this disease, which includes rollout of vaccination against the human papillomavirus (HPV) among girls [4].

HPV vaccines are safe and effective, and have achieved 90% efficacy in preventing cervical pre-cancers in young women naïve to the targeted HPV types [5–8]. The World Health Organization (WHO) recommends routine HPV vaccination for girls aged 9–13 years when feasible and sustainable, and where prevention of cervical cancer or other HPV-related diseases constitute a public health priority [9, 10]. The GAVI Alliance is supporting an HPV vaccine demonstration project in Kenya in 2013–2014 [11]. HPV vaccination presents unique challenges for implementation, given its three-dose series, protection against an infection that causes disease decades later, and target population of adolescent girls only [12, 13].

It is critical to understand communities’ vaccine-related perceptions and concerns to identify information, education and communication (IEC) needs for successful vaccine introduction [14–16]. Whereas formative research and pilot projects have been conducted in LMIC such as India, Peru, Vietnam, Uganda and other African countries [16]; there has been limited research in Kenya on HPV vaccine acceptability for young girls. Two available studies suggest that Kenyan mothers would be willing to accept a vaccine for their daughters to prevent cervical cancer, despite limited awareness of cervical cancer and other potential barriers, including concerns about side effects (infertility), cost, multiple doses and spouse disapproval [17, 18]. However, to date there has been no published research from Kenya on HPV vaccine acceptability among spouses (i.e., fathers of young girls) or community leaders, who may play critical roles in vaccine uptake [19, 20].

This study sought to explore sociocultural factors associated with HPV vaccine acceptability among opinion leaders and both male and female caregivers in a western province of Kenya. This article reports on caregivers’ and leaders’ cervical cancer and vaccination knowledge, attitudes and beliefs; and perceived acceptability of and barriers and facilitators to HPV vaccination, with implications for future HPV vaccine introduction.

## Methods

Qualitative research was conducted with caregivers and opinion leaders in three rural locations and one peri-urban location of Siaya County, Kenya, in July-August 2012. Siaya is among the poorest of Kenya’s 47 counties [21]. It ranks among the highest in child and maternal morbidity and mortality in all of Kenya [22], and has a higher HIV/AIDS prevalence rate compared to national averages [23, 24]. The study sites were located within a Health and Demographic Surveillance System (HDSS) established by the Kenya Medical Research Institute (KEMRI) and the U.S. Centers for Disease Control and Prevention (CDC), which covers a population of approximately 220,000. The study design was approved by the KEMRI Ethics Review Committee (Scientific Steering Committee #2167). This study adheres to the Qualitative Research Review (RATS) guidelines for reporting qualitative studies.

We conducted a total of five focus groups with caregivers (four groups of women and one of men) and 12 key-informant interviews with opinion leaders. Focus groups were used to prompt and observe in-depth, dynamic discussions among caregivers about cancer and vaccination; whereas interviews were intended to draw on the experiences and perceptions of community leaders. Open-ended, semi-structured discussion/interview guides were used to explore participants’ knowledge, attitudes and beliefs regarding cervical cancer and HPV vaccine acceptability for 9–12 year-old girls^a^. Interview guides were developed by CDC staff and revised with in-country project staff at KEMRI/CDC for cultural appropriateness and relevance. Caregiver focus groups were segmented by gender and led by a trained moderator, who was matched to participant gender to allow for open discussions of sensitive reproductive and sexual health issues. A greater number of focus groups were conducted with female caregivers since research suggests that mothers have primary responsibility for vaccine decision making; however, fathers may also be important household decision makers, whose perspectives impact a mother’s vaccination decisions [20]. The five focus groups allowed for a diversity of perspectives and to reach data saturation.

Caregivers were recruited by community mobilizers in three locations and through an ongoing parent-focused program (directed at improving HIV-prevention knowledge and communication skills) in the fourth location. Sampling was purposive, intended to gather a diversity of opinions among hard-to-reach caregivers in high-morbidity areas. Prospective participants attended a screening and consent session, during which they were informed about study objectives and participant rights and confidentiality; screened for eligibility; and consented if eligible. Participants were eligible if they reported being the caregiver of a girl aged 9–12 years and residing within the HDSS. Focus-group discussions were conducted in Dholuo (local language) and held at the chief’s camp of each location; they lasted two hours each.

Opinion leaders were recruited during a District Stakeholder Meeting and through referrals. Consent to participate was obtained prior to interviews, which were held at the location of each participant’s choosing. Each Interview lasted approximately one-and-a-half hours and was conducted in respondents’ language of choice.

### Data analysis

Focus group discussions were digitally recorded, transcribed and translated into English. A minimum of two team members reviewed transcripts to ensure the accuracy and completeness of translations against focus group notes and clarify any cultural references or direct translations. Transcripts were then analyzed using NVivo8 qualitative software. For interviews, detailed notes were taken and then reviewed by interviewer and note-taker to identify gaps and ensure accuracy and completeness. Notes were then coded and analyzed using concept matrices.

Grounded Theory method was used to analyze the data [25]. Four researchers independently reviewed the data and developed codes (themes) based on questions in interview guides and topics that emerged organically [26]. Using the codebook definitions, two researchers independently coded segments from the focus group transcripts and compared results. Through a group consensus process [25, 26], reliability was calculated by hand; coding discrepancies were discussed and reconciled, and revisions to the codebook were made. This process was repeated until a satisfactory level of agreement was reached. Reliability was checked at least one other time to ensure that coding remained consistent. The same consensus process was used for the interview notes. None of the researchers occupied dual roles.

## Results

### Characteristics of the sample

A total of 56 caregivers and 12 opinion leaders participated. Caregivers had an average of five children; most (79%) were married and one-quarter had not completed primary education (Table 1). In contrast, most (67%) opinion leaders had completed college or university; they represented leaders in health (n = 4), community relations (n = 3), and one each in immunizations, politics, religious organizations, education and media.

**Table 1:** **Focus group participant and key informant demographics**

	Focus groups participants (N = 56)			Key informants (N = 12)		
	n (%)	Mean (SD)	Range	n (%)	Mean (SD)	Range
**Age (years)**	–	38.3 (8.7)	18-62	–	44.6 (14.0)	24-67
**Gender**						
Female	44 (78.6%)	–	–	7 (58.3%)	–	–
Male	12 (21.4%)	–	–	5 (41.7%)	–	–
**Marital status**						
Married	44 (78.6%)	–	–	10 (83.3%)	–	–
Widowed	12 (21.4%)	–	–	1 (8.3%)	–	–
Single	–			1 (8.3%)		
**Education**						
No schooling	1 (1.8%)	–	–		–	–
Primary incomplete	14 (25.0%)	–	–	1 (8.3%)	–	–
Primary complete	16 (28.6%)	–	–	1 (8.3%)	–	–
Secondary incomplete	11 (19.6%)	–	–	2 (16.7%)	–	–
Secondary complete	13 (23.2%)	–	–	–	–	–
Tertiary/college	1 (1.8%)	–	–	5 (41.7%)	–	–
University	–	–	–	3 (25%)	–	–
**Location**						
East Alego	12 (21.4%)	–	–	1 (8.3%)	–	–
South Alego	12 (21.4%)	–	–	4 (33.3%)	–	–
S.E. Alego	11 (19.6%)	–	–	3 (25%)	–	–
Siaya Township	21 (37.5%)	–	–	4 (33.3%)		
**Number of children in home**						
Number of daughters	–	2.7 (1.3)	0-5	–	2.5 (1.8)	0-5
Number of sons	–	2.3 (1.7)	0-7	–	2.4 (1.6)	1-6
Age range of children within home (years)	–	–	3-34	–	–	3-26

### Awareness, knowledge, beliefs and perceptions

#### Cancer

Awareness of cancer was high among caregivers and opinion leaders. It was commonly described as an *infection, wound,* or *abnormal growth* affecting specific body parts. Participants regarded it as a serious, painful, and fatal disease that is incurable or difficult and expensive to treat. They acknowledged a sense of fear and shame associated with cancer, noting those affected “cannot freely socialize with others” and may be treated as outcasts and viewed as cursed.

### Cervical cancer and HPV

The majority of caregivers and opinion leaders had not previously heard of cervical cancer, though most recognized it, once explained by its symptoms, as a cancer of the female reproductive organs. Caregivers described it using local terms to refer to a *wound or sexually transmitted infection (STI) of the stomach or womb;* some confused it with other conditions in the genital or abdominal area, such as fibroids. By association with cancer, it was viewed as a serious and deadly disease for which treatment is expensive, inaccessible, unaffordable and ineffective. It was presumed to affect a woman’s ability to bear children.

Aside from health experts, participants were not aware of HPV or its link to cervical cancer, though most assumed cervical cancer was associated with sexual behavior or poor hygiene. A majority of focus groups believed a woman could become infected during sexual activity from her male partner’s *penile impurities*. Other presumed causes included having sex while menstruating, multiple partners, an STI that doesn’t heal, or a partner who is unfaithful; not bathing properly; and sexual initiation at a young age, particularly with an older man. Once informed about HPV, participants had many questions (Table 2), reflecting confusion about its causes and natural history, modes of transmission, symptoms, health effects, treatment and prevention in men and women.

**Table 2:** **Common questions from caregivers**

HPV	HPV vaccine
**Causes and natural history**	**Vaccine implementation**
• Where do people get HPV from?	• How and where will the vaccine be administered to girls?
• Is HPV hereditary?	• Will it be given to all girls in Kenya?
• Does HPV infect bodily fluids or blood?	• What other countries have implemented the vaccine?
• Does HPV only travel to the cervix (or does it affect other organs)?	
• What does HPV do to men?	**Vaccine benefits & alternatives**
	• How will it help us?
**HPV epidemiology**	• Is there another way of preventing cervical cancer?
• Does HPV mostly affect women?	
• If HPV cannot be seen, how do we know that most people have it at some point?	**Vaccine safety & side effects**
	• Has the vaccine been tested?
	• Is it being tested on our children?
**Modes of transmission**	• What are the side effects of this vaccine?
• Is it acquired through sexual intercourse?	• Does the vaccine affect future fertility?
• Are there other (non-sexual) ways you can get it?	
• Do men and women transmit HPV to each other?	**Vaccine effectiveness**
	• Will girls be prevented from cervical cancer after they are vaccinated?
**Symptoms and effects on the body**	• What is the duration of protection?
• What are the signs and symptoms of HPV?	• Will the vaccine work if it’s given to a girl who already has cervical cancer?
• How long does it take for them to appear?	• Will it still help if a girl misses a dose?
• How does one know if they are infected?	
• Could HPV turn into HIV?	**Age & gender concerns**
• Does HPV cause herpes?	• Why is it administered to girls aged 9–12 years, when cancer affects women (older)?
• Can HPV prevent a woman from getting pregnant, or having a healthy pregnancy?	• If most everyone has HPV then why are only children vaccinated and not adults? Why not boys?
• Does it cause other cancers or problems (e.g., prostate cancer in men)?	• Can the vaccine be given to girls aged 9–12 if they haven’t started monthly periods?

Participants reported diseases of the genital area were associated with shame and stigma. While caregivers did not believe cervical cancer to be common locally, several acknowledged it was not something people talk about, so it may silently impact their communities. They noted a cervical cancer diagnosis could cause blame or abuse between a woman and her husband. A majority of opinion leaders also noted women with cervical cancer may be unsupported or socially isolated from their communities, particularly if they cannot bear children.

*“It’s viewed like HIV and AIDS. If you have it, people don’t want to talk to you. You’re seen as an outcast in the community.” –* Opinion Leader

### Acceptability of a cervical cancer vaccine

With the exception of health and media experts (n = 3), no participants across focus groups or interviews had heard of the HPV vaccine. Once informed of a safe and effective vaccine against cervical cancer available for young girls, caregivers and opinion leaders were willing and eager to accept it. The majority of caregivers expressed trust in government decisions (recommendations) about vaccine safety. They were familiar with other childhood vaccines and believed them to be effective disease-prevention tools based on experiences seeing other diseases eradicated and vaccinated children growing up healthy.

*“There could be a change in the future of women… just like the case of cholera, nowadays it is unheard of.”* – Opinion Leader

The prevention of cancer for the future health of girls and communities was a primary motivator for vaccination across participant groups. The vaccine was seen as a way to protect children from a disease associated with pain, suffering and financial cost. Many cited a desire for their children to have a better future. Similarly, opinion leaders supported the vaccine as a life-saving and cost-reducing measure that could improve their communities’ social well-being. Preservation of a girl’s future fertility and averting cervical-cancer treatment costs were also motivators.

“*Cancerous infection is a disease that is greatly feared and if parents are well informed about it then they would not have a reason to oppose it*.” –Caregiver

A vaccine for girls only was seen as acceptable by caregivers, if communities are properly educated (e.g., that cervical cancer affects only women). Half of opinion leaders thought it would be ideal to vaccinate boys too, both to promote health equity and to maximize impact; some noted the possibility of stigmatizing or embarrassing girls by singling them out. Nonetheless, three-quarters of them agreed that vaccinating girls only was a reasonable public-health solution, given the high cost of vaccination, the country’s limited resources and the need to focus efforts where need is greatest.

*“I think, since it is called cervical cancer, meaning it is cancer that affects the reproductive system of women, it would be ok to vaccinate girls only.”* –Caregiver

Caregivers did not comment on the proposed age of vaccination but they acknowledged preadolescence as an age during which girls may have sexual risk. Several opinion leaders noted the proposed age seemed appropriate, though a minority (n = 3) were concerned the range was too narrow. They argued that some girls would need to be vaccinated earlier to receive protection prior to sexual debut, and older girls who had not yet had sex should still be able to benefit from vaccination. Whereas two opinion leaders suggested parents might be concerned about their vaccinated daughters becoming “careless” with sex, this concern was only voiced by one male caregiver*.*

### Questions and possible barriers to HPV vaccination

Caregivers were prompted for questions or concerns they would want addressed before vaccinating their daughters. Common questions (Table 2) related to vaccine safety and side effects (e.g., infertility), followed by vaccine effectiveness, age- and gender-related questions, the nature of the vaccine program, and vaccine delivery. Caregivers also expressed a desire to know more about cervical cancer, its consequences and impact on the community, and alternative prevention strategies.

Several themes emerged from discussions about potential fears and cultural/community barriers to vaccination, as described below.

1. *Lack of community awareness and education*
  Widespread ignorance about cervical cancer and the vaccine emerged as primary barriers to vaccination. Participants noted that the absence of education could result in low demand for vaccination, and potentially damaging rumors or misconceptions could fill the gaps in knowledge.
2. *Cultural Beliefs*
  Participants across groups identified cultural beliefs as potential challenges to vaccination, particularly among the elderly, traditional herbalists and some men/fathers, who distrust vaccines or do not believe in preventive health services; and specific religious groups that reject modern medicine. Two focus groups discussed beliefs that vaccines cause more harm than good, including prolonged crying and fever in the short term. According to two opinion leaders, some people believe vaccinations are small injections of disease causing disease or death. However, these cultural attitudes were thought to be held by a minority and amenable to change with education.
3. *Adverse events*
  Participants noted adverse events and side effects, including pain or physical reactions, could discourage vaccine series completion. Opinion leaders feared rumors could spread from real or perceived adverse events, and that negative experiences, such as long wait times or rude staff, could also inhibit vaccine uptake.
4. *Access Challenges*
  Challenges reaching girls with three doses of a vaccine, including transportation, demands on time and competing priorities were noted, particularly for clinics, since long travel distances may be required. Girls may also be reluctant to visit clinics, with aversions from past experience of injection pain and suboptimal care. Whereas school settings might conveniently reach a majority of girls, this would miss out-of-school girls and girls who are absent from school on the day of vaccination.
5. *Stigma*
  Opinion leaders noted that sexual health may be taboo and parents may struggle to talk to their children about prevention of cervical cancer. They also expressed concern that girls could be presumed sick (e.g. HIV-positive) and stigmatized if seen making multiple visits to a health center. Similarly, caregivers felt that school or community settings could present privacy challenges, introducing the potential for embarrassment or ridicule of girls by their peers due to presumed associations with puberty, participation in a research study, or illness/HIV.
6. *Fears, Rumors and Distrust*
  A lack of information, coupled with distrust of the medical community held by a minority of community members, could lead to concerns or rumors about the intent of the vaccination effort. A few opinion leaders noted that communities might fear that their daughters are being used for research or distrust the intent of “whites” or foreign agencies supporting the program. Concerns about infertility (as a side effect) or suspicions about a covert sterilization effort were mentioned by half of opinion leaders and in two focus groups, though sterilization concerns were more predominant among male caregivers. Targeting young girls for vaccination rather than older women, who are affected by the disease, could fuel these suspicions.

*“There are people in the community who are fond of spreading rumors. They may inform the parents that their children are only being used for research purposes and the vaccine would be of no help to their children.”* – Caregiver

### Facilitators to vaccination

Community mobilization and sensitization were seen as critical for raising cervical cancer awareness; prompting demand for vaccination; and helping to overcome stigma. Participants discussed the need to educate mothers and fathers (as family-level decision makers), as well as girls. They suggested promoting community dialogue and education through organizations and events (e.g., Chief’s meetings, schools, churches, funerals, workshops, awareness rallies/walks, concerts); champions (e.g., cervical cancer survivors, affected families, health care providers); and mass media, advertising and print information. Opinion leaders suggested approaching communities in an “ethical” manner, educating them based on existing beliefs and addressing concerns in culturally sensitive ways through leaders (e.g., chiefs, community mobilizers, health workers, teachers and school administrators). Most believed potential “resisters” would be amenable to vaccination, if approached with culturally appropriate education from traditional leaders (e.g., registered herbalists, religious leaders).

*“Cervical cancer is like a new thing…We need to face realities. Find an approach to reduce stigma so we can address the disease. Let us not hide from it because we are in denial.”* –Opinion Leader

Other facilitators included convenient, safe and accessible community settings for vaccination that assure patient privacy; competent, trusted and friendly staff; and endorsement by government, hospitals and medical institutions. Finally, experts emphasized the importance of leveraging lessons learned from past programs, including having a government policy in place to support vaccination and integrating vaccination into other health services (e.g., school health or community outreach programs).

*“Once you have a policy in place, they [parents] follow the policy.”* – Opinion Leader

## Discussion

This research sought to explore factors associated with HPV vaccine acceptability among caregivers and opinion leaders in Kenya to inform communication efforts in preparation for vaccine introduction. Although cancer was a widely known and feared disease, cervical cancer was not commonly recognized. It was understood generally in terms of its symptoms, as a disease that is linked to sexual risk factors [20, 27] and infertility. It was feared and seen as shameful; something that is not openly discussed. Overall acceptability of a vaccine that prevents cervical cancer was high, if endorsed by trusted government agencies and if communities are properly sensitized.

Our findings are similar to those in other LMIC where there is limited knowledge of cervical cancer, a general awareness and high perceived severity of cancer, and high acceptability and perceived value of vaccines for disease prevention [18, 20, 27, 28]. They underscore the need for mass-media and community efforts to raise cervical cancer awareness [17, 18] and address stigma for successful vaccine introduction in Kenya. Visible endorsement by government leaders [19, 29] and support from community influencers (e.g., chiefs, community health workers, teachers/school administrators) [30], as well as both parents, will be critical for uptake [17, 19]. Involvement of leadership and community champions may also be critical for combatting stigma and making cervical cancer relevant to Kenyan communities [31, 32].

A well-planned and funded strategic communication/mobilization plan can help reach and maintain vaccine coverage [33]. In Kenya, an effective strategy should not only address cervical cancer awareness, stigma and the need for vaccination, but issues of vaccine safety, efficacy and delivery, including potential concerns regarding its effect on fertility and intent of the program [20, 27, 28, 34] (Table 3). Such concerns may be prompted by the vaccine’s focus on young girls, its perceived ‘newness’ , and its origination in high-income countries [25, 26]. Rumors or confusion could be particularly important to address in vaccine demonstration projects, which could be misunderstood as research trials. Trusted leaders can clarify misconceptions and provide assurances of vaccine safety, program goals and vaccination benefits [20, 27, 35]. Trained mobilizers can help contain resistance from individuals or groups, which can negatively impact community acceptance [12].

**Table 3:** **HPV vaccine communication objectives**

***Raise cervical cancer awareness & understanding***	• Cervical cancer is a serious disease that affects a woman’s cervix.
	• It often strikes women in their 30’s and 40’s. But it starts to develop many years earlier.
	• It is one of the most common cancers in women worldwide and a main cause of cancer death.
	• Each year in Kenya, about 2,500 women get cervical cancer and 2,000 women die from it.
***Create demand for HPV vaccine***	• A vaccine can prevent girls from getting cervical cancer later in life. It is given as an injection in the arm. Three doses are needed for full protection.
***Address safety concerns***	• It is best if the vaccine be given to girls around the ages of 9–13 years. Vaccinating girls at this age ensures that they are protected long before cervical cancer begins to develop.
***Address concerns about sterilization***	• It is best if the vaccine be given to girls around the ages of 9–13 years. Vaccinating girls at this age ensures that they are protected long before cervical cancer begins to develop.
***Build on positive vaccine perceptions and emphasize perceived benefits***	•The vaccine protects the reproductive health of girls/women.
	o By vaccinating young girls, we can protect their health and their future – so they can be around for their own children and families.
	o Prevention is better than treatment or cure.
***Create realistic expectations of vaccine to help minimize rumors or false expectations***	• The most common side effects are pain, redness or swelling in the arm. Some girls may get a mild fever, headache, or nausea.
	• The vaccine does not protect against HIV or STDs such as chlamydia or gonorrhea.
***Address health equity concerns***	• The vaccine is intended primarily for prevention of cervical cancer, a disease that does not affect men. But everyone can help prevent cervical cancer.
	o Parents can take their daughters to get vaccinated.
	o Women can get screened for cervical cancer (Pap test or visual inspection) so that early problems can be found and treated – even before cancer develops.
	o Men can support their wives, sisters and mothers in getting screened for cervical cancer.
	o Everyone can start talking openly about cervical cancer and educating others about prevention.

Gain-framed messaging (i.e., promoting the benefits of vaccination, rather emphasizing the consequences of *not* getting vaccinated), which builds on positive perceptions of vaccines, may be most effective in promoting HPV vaccination [20, 27, 36] and help minimize stigma [37]. Implementers should consider marketing the vaccine as a “cervical cancer vaccine” [20, 27, 28, 34] that promotes reproductive health, since cancer is a familiar disease that people are motivated to prevent and reproductive health is highly valued in Kenya. In contrast, HPV generated much confusion among caregivers, distracting from the issue of cervical cancer.

The proposed age of vaccination did not prompt caregiver concerns about sexual disinhibition, as it has elsewhere [27, 35]. However, some opinion leaders expressed health-equity concerns, preferring that all females (young and old), as well as boys, have access to vaccination [28]. Additionally, the singling out of girls was regarded as potentially stigmatizing or embarrassing for them. These challenges can be addressed (at least in part) with communication and social marketing strategies, including: (a) raising awareness that cervical cancer only affects women; (b) marketing vaccine delivery as a *package* of cervical-cancer prevention tools, which also includes screening for women; and/or (c) integrating HPV vaccination into other health outreach or school-health programs so that girls are vaccinated as part of broader public-health interventions rather than singled out. Integration into school health efforts has been effectively achieved in other African countries [19, 29, 38], though cost and delivery challenges may impede the long-term sustainability of such efforts and there is a need for further delivery strategies to reach girls not in school, who may be in greatest need [39]. A combination of school-based and health facility-based delivery methods may therefore offer the greatest promise for effectively reaching girls with HPV vaccination in LMICs such as Kenya [40]. These programs could be supported by text-based reminders or other digital media tools to promote series completion [41]. To realize this promise, however, critical infrastructure, service quality and delivery challenges will need to be addressed— from staff training to cold chain and reporting systems [42]. GAVI demonstration projects are currently underway to identify the best, most affordable and sustainable HPV vaccine delivery strategies in LMICs with weak health systems or immunization programmes [43].

## Conclusion

This study is the first to assess HPV vaccine acceptability among both male and female caregivers and opinion leaders in Kenya. It underscores the need for adequate planning and resources for IEC prior to vaccine introduction to raise awareness of cervical cancer, prompt demand for vaccination, and minimize stigma and potential resistance. These results are based on a small sample of caregivers and opinion leaders (from limited fields of expertise and geographic areas) recruited through KEMRI/CDC and may not be generalizable. It is possible that participants were influenced by social desirability (given that the research was conducted by a health agency), or that they differed in their opinions on vaccination and public-health institutions from caregivers and opinion leaders who did not participate in the study. Details on those who chose not to participate (and why) were not collected, which is a study limitation. However, results are intended to be directional in nature, informing future efforts. Future evaluations should include caregivers and opinion leaders from different regions and cultural/ethnic affiliations, including multi-level school and policy leaders and experts in cancer prevention, immunization, STI prevention and adolescent health, as well as young girls themselves. Such research should be supported by health-systems and policy research to assess capacities, structures, processes and policy environments for vaccine implementation.

## Endnote

^a^Parents of 9–12 year-old girls were selected for this research because this (9–12 years) was the tentatively targeted age range for HPV vaccination efforts in Kenya at the time this research protocol was developed.
